# Does Compact Built Environment Help to Reduce Obesity? Influence of Population Density on Waist–Hip Ratio in Chinese Cities

**DOI:** 10.3390/ijerph17217746

**Published:** 2020-10-23

**Authors:** Chun Yin, Bindong Sun

**Affiliations:** 1 Research Center for China Administrative Division, East China Normal University, Shanghai 200241, China; cyin@geo.ecnu.edu.cn; 2 Institute of Eco-Chongming, Shanghai 202162, China; 3 Future City Lab, East China Normal University, Shanghai 200241, China

**Keywords:** compact development, obesity, population density, China, fixed-effect model

## Abstract

This study aimed to identify the non-linear association between population density and obesity in China and to provide empirical evidence for the public health orientated guideline of urban planning. By conducting a longitudinal study with data collected from the China Health and Nutrition Survey (CHNS) between 2004 and 2011, we applied fixed-effect models to assess the non-linear association between the compact built environment and waist–hip ratio (WHR), controlling for sex, age, nationality, education, employment status, marital status, household size, household income, and residents’ attitudes. Our findings reveal that the built environment is one of the key determinants of obesity. The U-shaped influence of population density on WHR was observed. Moreover, influence differs according to sex and weight status. Our findings indicate healthy city planning has the potential to improve the built environment to reduce obesity risk and promote public health.

## 1. Introduction

Obesity is a major risk factor for many diseases, such as type-2 diabetes, sleep apnea, high blood pressure, some kinds of cancers, etc. [1]. In the past 40 years, obesity rates have risen dramatically worldwide [2]. By 2016, more than half a billion people were obese, and adult obesity rates in China had increased to 6.2%, up from 2.4% in 2000 [3].

Many factors contribute to obesity, such as genetics, diet, physical activity, socioeconomic status (sex, age, race, income, etc.), psychological factors, culture, environmental factors, and so on [4]. Among them, the built environment plays an important role in shaping obesity [5]. Many studies from developed countries like North America and Europe show that the compact built environment is negatively associated with obesity [6], because people living in compact neighborhoods are more likely to travel by active modes and have higher availability of health food [7,8]. Therefore, the World Health Organization recommends increasing compactness to prevent obesity and obesity-related chronic diseases [9,10,11].

However, the association between compactly built environment elements and weight status is context-sensitive [12]. Many studies have examined the association between population density and obesity, but their findings are mixed [13,14]. Most studies based on the North American and European context found that population density has a negative association with obesity, but evidence from China revealed that people living in denser neighborhoods tended to have a higher risk of obesity [15]. Differing from western developed countries, Chinese cities are much more compact and dense [16]. Taking population density as an example, the average city population density in China was about nine times higher than that of the American cities in 1995 [17]. Over-compactness results in negative environmental externalities, such as air pollution, noise, and loss of green space [18], which are harmful to residents’ health and lead to an increase in the risk of obesity [16]. Therefore, the total effect of compactness should be the trade-off between positive effects and negative effects. That is to say, the association between population density and obesity might be a non-linear rather than a linear relation. However, most of the existing literature ignored the non-linear effect of compactness on obesity.

Moreover, previous studies paid less attention to inferring causality. This is because most of them failed to control for unobservable but time-invariant variables, such as genetics, culture, etc., as they did not employ a longitudinal design. In addition, many previous studies ignored self-selection effects, which means that people tend to choose residential locations and carry out daily activities based on their travel preferences or attitudes [19]. For example, if people treat daily physical activity as an important thing, they might choose to live in neighborhoods with ample physical facilities, meaning that they partake in physical activities more and thus have a lower weight status. In this case, it is not physical facilities but personal attitudes that have a true and direct influence on health outcomes. To deal with the endogeneity, we applied fixed-effects models based on panel data (also called longitudinal data) to control unobservable but time-invariant variables and controlled residents’ attitudes in the models directly to reduce self-selection effects.

Overall, more robust evidence about the causal relationship between population density and obesity should be provided, given that the non-linear causal effect of population density on obesity is far less explored. To fill this gap, we examined the causal association between built environment and obesity in China, particularly focusing on the non-linear effect of population density, its heterogeneity of sex and weight status, and its influential paths, based on fixed-effects models.

## 2. Materials and Methods

### 2.1. Data Source

Data came from the China Health and Nutrition Survey (CHNS) [20,21], which is an open cohort, international collaborative project, collected in 1989, 1991, 1993, 1997, 2000, 2004, 2006, 2009, 2011, and 2015. This survey applied a rigorous multistage, random cluster process to draw a sample of over 30,000 individuals in 15 provinces and municipal cities that vary substantially in geography, economic development, public resources, and health indicators. Moreover, community data were collected in surveys of food markets, health facilities, etc. One of the aims of this survey was to understand how the transformation of Chinese society is affecting the health and nutritional status of its population (for more information please refer to https://www.cpc.unc.edu/projects/china).

### 2.2. Outcome Variable: Waist–Hip Ratio

Waist–hip ratio (WHR) was used to measure obesity in this study, because it is a more accurate indicator of visceral adiposity and obesity-related health risks than body mass index, which fails to distinguish between fat and lean body mass and does not account for fat distribution [13,22,23,24]. According to the World Health Organization (WHO), abdominal obesity is defined as a waist–hip ratio above 0.9 for men and above 0.8 for women for Asians [25]. In this study, the mean of WHR is 0.87, and the mean of men and women is 0.89 and 0.85, respectively, thus suggesting that abdominal obesity cannot be ignored in China.

### 2.3. Built Environment Elements

The key explanatory variable in this study is the neighborhood population density. It is a proxy for compact-built communities because it is one of the most important elements of compactness [26,27] and is also highly associated with other elements of compactness [18]. Dense neighborhoods usually tend to have mixed land uses, well-connected streets, high access to destinations, and intensive transit services [28,29]. On the other hand, higher population density also serves as a proxy for poor areas, congestion, limited activity space, and air pollution [30,31,32].

Table 1 presents the descriptive statistics of each built environment element. We used both continuous and categorical population density variables to explore the non-linear effect of population density on WHR. The food environment is measured by grocery density and restaurant types, indicating healthy and junk food availability, respectively. Physical activity environmental elements include the presence of parks, the presence of bus stops, company density, and the presence of schools. Parks are one of the most important destinations for residents when it comes to doing physical activity. Bus stops could encourage active travel instead of driving and increase the level of physical activity. Higher company density provides more job opportunities for residents, and thus it promotes the jobs-housing balance and reduces the auto commute. Vehicles around schools usually have lower speeds, which provides a safer environment for physical activity. All of these physical activity environment elements might reduce WHR by encouraging physical activity.

### 2.4. Confounding Variables: Respondents’ Characteristics

Table 1 also shows the descriptive statistics of respondents’ characteristics, which are controlling variables in this study, including socio-economic attributes, attitudes, and survey years. Socio-economic attributes include sex, age, nationality, education, employment status, marital status, household size, and household income. Attitude variables reflect whether the respondent thinks that having a healthy diet or being physically active is important.

### 2.5. Mediators: Respondents’ Behaviours and Health Status

Some variables influence WHR, and are also outcomes of built environments, so we treated them as potential mediators between built environments and WHR. There are two types of mediators. One reflects behaviors and habits, including smoking, drinking, car ownership (a proxy of car use), sleep duration, physical activity duration, and sedentary duration. The other is the health status; we used a dummy variable indicating if the respondent is sick. Since these variables are potential mediators, we only controlled them in the robust models, and also examined their mediation effects between population density and WHR.

### 2.6. Data Process

We chose four waves of survey data collected in 2004, 2006, 2009, and 2011, because they included all exposure, outcome, and confounding variables of interest for this study. Given the huge built environment differences between urban areas and rural areas in China, we only explored the relationship between the built environment and obesity in urban areas. Considering the difference in WHR among teenagers, adults, and seniors [13], we focused on the residents who were 18 to 65 years old at the baseline year (2004) and included the respondents who were involved in the four waves. Based on these criteria, the number of respondents was 3448 at the baseline year, then 2975, 2368, and 1882 respondents were followed up in 2006, 2009, and 2011, respectively. By deleting observations without WHR and density information, we finally got unbalanced panel data with 5479 observations. The percentage of missing values is 27%.

Given that residents’ sex, nationality, age, education, job, marriage status, and most of the neighborhood areas are usually unchanged or change regularly in China, we filled in the missing values in the data according to the last observation carried forward. When the last observation carried forward was also missing, we filled in the missing values in the data based on the next observation carried backward. The reason is that for the longitudinal data, investigators usually would not always record the information of the same respondent. To reduce possible effects of outliers, we winsorized the WHR, sleep duration, physical activity duration, and sedentary duration at the 0.5th and 99.5th percentiles.

### 2.7. Statistical Models

We applied fixed-effect models to explore the causal relationship between the built environment and WHR. Equation (1) shows the baseline model:

(1) $$WHR_{it}=\alpha_{0}+\alpha_{1}BE_{it}+\alpha_{3}SES_{it}+\alpha_{4}ATT_{it}+T_{t}+\gamma_{i}+\varepsilon_{it}$$

*WHR_it_* is the waist–hip ratio for individual *i* in the survey year *t*. *BE_it_*, *SES_it_*, and *ATT_it_* represent the matrices of built environment elements, socio-economic attributes, and personal attitude variables for individual *i* in the survey year *t*, respectively. *T_t_* is the survey year used to control for time fixed effects. *γ_i_* is the individual fixed effect to control for time-invariant unobservable variables that vary across individuals. Moreover, we analyzed the heterogeneous effects of the built environment on WHR across sex and weight status with subsamples because of the sex difference in the deposition of body fat and the different cut-off points of abdominal obesity between men and women [25]. Moreover, we used clustered robust standard errors to correct the bias resulting from the fact that people living in the same neighborhood are affected by the same neighborhood built environment [33]. All models were run in STATA 14 with the xtreg command. To confirm the effect size of each variable, we report standardized regression coefficients.

To understand how the built environment affects WHR, we tested some mediators in terms of whether they carry the influence of the built environment to WHR according to the Sobel test [34]. At first, we added all mediators into the baseline model:

(2) $$WHR_{it}=\alpha_{0}+\alpha_{1}BE_{it}+\alpha_{3}SES_{it}+\alpha_{4}ATT_{it}+M_{it}+T_{t}+\gamma_{i}+\varepsilon_{it}$$

where *M* is the mediator. If a mediator affected WHR significantly, we examined the effect of the built environment on the mediator:

(3) $$M_{it}=\alpha_{0}+\alpha_{1}BE_{it}+\alpha_{3}SES_{it}+\alpha_{4}ATT_{it}+T_{t}+\gamma_{i}+\varepsilon_{it}$$

Following this, if both the built environment and the mediator had a significant effect on WHR, we conducted the Sobel test [34]:

(4) $$Z=\frac{ab}{\sqrt{b^{2}s_{a}^{2}+a^{2}s_{b}^{2}}}$$

where *a* and *b* are unstandardized regression coefficients for the association between the independent variable and the mediator and the association between the mediator and the dependent variable, respectively. The *s_a_* and *s_b_* represent the standard error of *a* and *b*, respectively. Only the mediator passing the Sobel test can be a mediator between the built environment and *WHR*.

## 3. Results

### 3.1. The Association between Built Environment and WHR

Table 2 shows the estimates of the causal association between built environment elements and WHR, and also presents the heterogeneous effects of the built environment on WHR across sex and weight status. We used both the continuous population density variable and the categorical population density variable to estimate the non-linear effect of population density on WHR.

**Figure 1:**
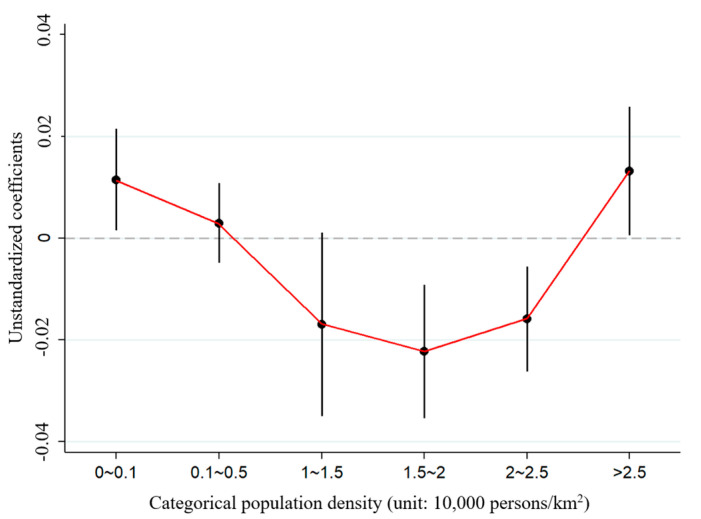
The U-shaped effect of population density on waist–hip ratio. Note: The vertical lines represent 95% confidence intervals of the coefficients.

Continuous population density is not related to WHR, but categorical population density shows a U-shaped relation with WHR (Figure 1). WHR first decreases as population density increases from 0 to 20,000 persons per km^2^. When population density is between 20,000 and 25,000 persons per km^2^, the increasing effect of high population density on WHR appears, though the decreasing effect still overwhelms the increasing effect. Beyond this range (population density above 25,000 persons per km^2^), WHR increases, suggesting that extremely high population density would increase the risk of obesity [13]. Given that the effect of population density on WHR is a result of balancing the increasing effect and the decreasing effect of population density, the insignificant association between continuous population density and WHR might result from the fact that the positive and negative effects cancel each other out [13,35].

The relationship between population density and WHR and its threshold differ in sex and weight status. In terms of women, when population density is between 10,000 and 20,000 persons per km^2^, it is only negatively associated with obese women’s WHR. When population density is above 20,000 persons per km^2^, it would increase non-obese women’s WHR significantly. Regarding men, the U-shaped curve only exists in the non-obese weight status group. When population density is below 25,000 persons per km^2^, it has a negative association with non-obese men’s WHR. Beyond this threshold, population density tends to increase non-obese men’s WHR. We did not find evidence to support the association between population density and obese men’s WHR.

In terms of other built environment variables, the food environment matters for WHR. Grocery density is negatively associated with WHR, particularly for non-obese men, while the restaurant types have a positive association with WHR in the full sample, consistent with previous studies [36]. Among the physical activity environmental variables, the presence of bus stops exhibits a significant negative association with WHR, consistent with previous studies [37], while the other physical activity environmental features are not significantly associated with WHR. Indeed, the latter is also consistent with some previous studies, which found that access to parks and fitness facilities was only associated with physical activity but not with body composition or obesity [38,39,40].

Additionally, among socio-economic attributes and attitudes, only marriage status is associated with WHR. Married respondents have higher WHR than those unmarried respondents, which is consistent with previous studies [41,42]. A possible reason is that married people exercise less [43].

### 3.2. The Mediators between Built Environment and WHR

To identify the mediators between the built environment and WHR, we added all potential mediators into the continuous and the categorical population density models, respectively (Table 3). We found that car ownership and sedentary duration are significantly positively associated with WHR, but physical activity duration has a significantly negative association with WHR, and other potential mediators are insignificant. We then examined whether the built environment affects the three significant mediators one by one in both continuous and categorical population density models. However, the results show that the built environment is only associated with physical activity duration in the continuous population density model. Population density has negative association with physical activity duration, but grocery density is positively associated with physical activity duration. Sobel tests identify that physical activity duration is a mediator both between population density and WHR (*Z = 2.055, p =* 0.04) and between grocery density and WHR (*Z =* −2.150, *p =* 0.03). That is to say, higher population density increases WHR by reducing physical activity duration, and higher grocery density decreases WHR by increasing physical activity duration.

## 4. Discussion

By employing fixed-effect models with clustering at a neighborhood level based on a CHNS dataset, we identified the causal relationship between the built environment and WHR, and found that population density has a non-linear influence on WHR.

The non-linear relationship between population density and health outcomes was discussed in several studies [44,45], but empirical evidence is far from sufficient. We found the U-shaped curve between neighborhood population density and WHR, and its turning point is 25,000 persons per km^2^, providing empirical evidence in the Chinese context. The U-shaped curve is consistent with previous findings in China [13,15]. For example, based on a sample of 4114 individuals in 2014 from across China, a study found a U-shaped curve between population density at the city level and the body mass index [15]. However, their results show that only city population density instead of neighborhood population density is associated with obesity. In the UK, a study also explored the non-linear relationship between residential density and WHR by using cross-sectional data for adults aged 37 to 73 years old. It found that the relationship between density and obesity was inverted U-shaped, in direct contrast with our finding, and the turning point was 1800 units of dwelling per km^2^ [46]. However, this study measured density by dwelling density, which is smaller in dense central areas because it ignores the household size [47]. Moreover, the cross-sectional analysis failed to capture the causal association and potential pathways of population density. Overall, none of the previous studies identified the U-shaped causal association between neighborhood population density and WHR, as well as the influential pathways of this association.

For the neighborhoods with a population density lower than 25,000 persons per km^2^, increasing neighborhood population density could reduce WHR. It is consistent with findings from both western cities [48] and Chinese cities [16,49]. One possible reason for this is that higher population density might shorten travel distance and promote non-motorized travel [13]. We did not find that car ownership is a mediator between population density and WHR, but found that car ownership does have a positive association with WHR.

However, population density is positively associated with WHR when it is above 25,000 persons per km^2^. Limited by data availability, we only found that the physical activity duration is the mediator in this study. That is, the increase of population density reduces physical activity and leads to a higher risk of obesity [32,50]. There are several possible reasons for this. First, the sizes of per capita public and green spaces, which are usually are used for doing physical activity, tend to decrease. Second, although dense residential neighborhoods could encourage non-motorized travel, influence on modal shift is limited in over-compact neighborhoods [16,47]. Additionally, extremely high population density implies a hectic lifestyle and life stress has a positive association with the risk of obesity [51,52]. Furthermore, higher population density is positively associated with air pollution, which is a main correlate of obesity [53,54].

The limitations of this paper originate from data unavailability. First, we could not examine more influence pathways between population density and obesity, such as stress, pollution, the area of green spaces, calorific intake, and energy consumption, etc. Second, some variables are crude in this study and might lead to estimating bias. For example, in theory, higher population density could reduce driving, and thus reduce the risk of obesity. However, in this study, we could only treat car ownership as a proxy for driving due to data unavailability. Moreover, most of the built environmental elements were measured by the presence and density, as there was a lack of measurements for their proximity and distribution pattern.

Despite these limitations, the strengths of the present study are obvious. First, it is the first study to have identified the U-shaped causal relationship between population density and obesity and its influential path. Many developed countries in North America and Europe have relatively lower population density and are facing low-density urban sprawl; thus, among these studies, most found that only increasing population density could reduce the risk of obesity. However, China has large areas, and the development of different regions is unbalanced at the same time, thus meaning there are both low and (extremely) high population density neighborhoods in China. Therefore, based on the Chinese specific context, we could find the U-shaped curve law between population density and obesity across a great range of neighborhood densities. Second, by applying fixed-effects models based on panel data, we controlled many time-invariant unobservable variables, which meant that our models were more effective in inferring the causal relationship between built environment and obesity than the previously-used models based on cross-sectional data. We also controlled people’s attitudes towards a healthy diet and physical activity, to reduce the bias from self-section effects, which have often been ignored by previous studies.

## 5. Conclusions

This study is the first to apply fixed effects models to identify the causal association between the compact built environment and obesity in the Chinese context, particularly focusing on the non-linear effect of population density and its pathways and heterogeneity, based on the China Health and Nutrition Survey in 2004, 2006, 2009, and 2011. After controlling residents’ socio-demographic attributes, self-selection, and other confounding variables, we found that the population density shows a U-shaped relationship with waist–hip ratio. Therefore, these results have important implications when it comes to planning human health-friendly cities. First, this study shows that the built environment does affect residents’ obesity status, and thus urban planning has the potential to promote public health by improving the built environment. Second, planners should densify less populated neighborhoods, and at the same time establish a density limit to prevent the development of extremely dense neighborhoods. Third, planners should also pay attention to improving access to stores of healthy food and public transit. Last but not least, planners should consider the different effects of the built environment on different sex residents’ health status when they are planning neighborhoods.

## Figures and Tables

**Table 1:** The definition and descriptive characteristics of the built environment, socio-economic attributes, respondents’ attitudes, and possible mediators.

Variable	Definition	Obs.	Mean (SD)/Obs (%)	Min	Max
built environment					
Population density (Cont.)	A continuous variable indicating the neighborhood population density, which is population size divided by neighborhood area (unit: 10,000 persons/km^2^)	5479	0.82 (2.25)	0	16.83
Population density	A categorical variable indicating the neighborhood population density (unit: 10,000 persons/km^2^). (0,0.1] = 1; (0.1,0.5] = 2; (0.5,1] = 3 (reference); (1,1.5] = 4;(1.5,2] = 5; (2,2.5] = 6; more than 2.5 = 7	5479		1	7
0~0.1			2251 (41.08%)	0	1
0.1~0.5			1722 (31.43%)	0	1
0.5~1			610 (11.13%)	0	1
1~1.5			340 (6.21%)	0	1
1.5~2			106 (1.93%)	0	1
2~2.5			133 (2.43%)	0	1
>2.5			317 (5.76%)	0	1
Food environment					
Grocery density	The number of fruit/vegetable stores and vendors in the neighborhood divided by neighborhood area (unit: number/km^2^)	5479	12.68 (51.60)	0	700
Restaurant types	The number of restaurant types in the neighborhood, including fast-food restaurants, other indoor restaurants, outdoor fixed food stalls, and ice cream parlors	5479	2.01 (1.14)	0	4
Physical activity environment					
Presence of parks	A dummy variable indicating whether parks are in the neighborhood	5479	1450 (26.46%)	0	1
Presence of bus stops	A dummy variable indicating whether bus stops are in the neighborhood	5479	4033 (73.61%)	0	1
Company density	The number of private enterprises in the neighborhood divided by neighborhood area (unit: number/km^2^)	5479	10.60 (61.90)	0	803.57
Presence of schools	A dummy variable indicating whether schools are in the neighborhood	5479	3711 (67.73%)	0	1
Socio-economic attributes					
Male	A dummy variable indicating whether the respondent is male	5479	2632 (48.04%)	0	1
Age	The respondent’s age	5479	47.98 (12.06)	18	72
Han nationality	A dummy variable indicating whether the respondent is Han nationality	5479	4992 (91%)	0	1
Education	The year of respondent’s education	5479	8.71 (4.04)	0	18
Employment	A dummy variable indicating whether the respondent has a job	5479	2896 (52.86%)	0	1
Married	A dummy variable indicating whether the respondent is married	5479	4800 (87.61%)	0	1
Household size	The number of family members living together	5479	3.52 (1.36)	1	10
Household income	The logarithm of household annual gross income (unit: 10,000 yuan)	5479	10.11 (1.38)	0	13.97
Attitudes					
Healthy diet	A dummy variable indicating the importance of eating a healthy diet is very important or the most important	5479	1899 (34.66%)	0	1
Physically active	A dummy variable indicating whether the importance of being physically active is very important or the most important	5479	1666 (30.41%)	0	1
Mediators					
Smoker	A dummy variable indicating whether the respondent smokes	5472	1809 (33.06%)	0	1
Drinker	A dummy variable indicating whether the respondent drinks	5477	2138 (39.04%)	0	1
Car ownership	A dummy variable indicating whether the respondent owns one or more cars	5474	508 (9.28%)	0	1
Sleep duration	Respondent’s sleeping duration per day (unit: hour)	5417	7.83 (1.17)	4	14
Physical activity duration	Respondent’s physical activity duration per week (unit: hour)	5479	0.95 (3.08)	0	17.5
Sedentary duration	Respondent’s sedentary duration per week (unit: hour)	5479	21.90 (14.89)	0	85
Sick	A dummy variable indicating whether the respondent is sick	5466	813 (14.87%)	0	1

**Table 2:** Standardized estimates of the effects of built environment elements on waist–hip ratio (WHR) and heterogeneous effects across sex and weight status.

	Continuous Population Density		Categorical Population Density		Women				Men			
					Non−Obese WHR (WHR < 0.8 ^a^)		Obese (WHR ≥ 0.8 ^a^)		Non−Obese WHR (WHR < 0.9)		Obese (WHR ≥ 0.9)	
	Beta	CI	Beta	CI	Beta	CI	Beta	CI	Beta	CI	Beta	CI
Population density (Cont.)	0.031	[−0.001,0.003]										
Population density												
0~0.1			0.077 *	[0.001, 0.021]	0.048	[−0.010, 0.015]	0.112	[−0.000, 0.027]	0.074	[−0.006, 0.019]	0.162	[−0.005, 0.035]
0.1~0.5			0.019	[−0.005, 0.011]	0.078	[−0.003, 0.013]	0.010	[−0.007, 0.009]	0.081 *	[0.000, 0.014]	−0.012	[−0.013, 0.011]
0.5~1 (ref.)												
1~1.5			−0.056	[−0.035, 0.001]	−0.026	[−0.019, 0.011]	−0.122 ***	[−0.047, −0.009]	0.045	[−0.005, 0.022]	−0.024	[−0.028, 0.020]
1.5~2			−0.042 **	[−0.035, −0.009]	0.139	[−0.006, 0.061]	−0.050 **	[−0.039, −0.006]	0.016	[−0.008, 0.019]	−0.036	[−0.031, 0.010]
2~2.5			−0.033 **	[−0.026, −0.006]	0.174 **	[0.009, 0.041]	0.000	[−0.017, 0.018]	−0.061 ***	[−0.027, −0.009]	−0.037	[−0.032, 0.008]
>2.5			0.042 *	[0.001, 0.026]	0.134 **	[0.005, 0.025]	0.076	[−0.001, 0.038]	0.141 **	[0.008, 0.047]	−0.060	[−0.027, 0.002]
Food environment												
Grocery density	−0.047 **	[−0.000, −0.000]	−0.051 ***	[−0.000, −0.000]	−0.009	[−0.000, 0.000]	−0.041	[−0.000, 0.000]	−0.042 ***	[−0.000, −0.000]	0.003	[−0.000, 0.000]
Restaurant types	0.068 *	[0.001, 0.008]	0.072 *	[0.001, 0.008]	−0.002	[−0.003, 0.003]	0.033	[−0.002, 0.006]	0.039	[−0.003, 0.005]	0.095	[−0.000, 0.008]
Physical activity environment												
Presence of parks	0.018	[−0.006, 0.012]	0.019	[−0.005, 0.012]	−0.022	[−0.008, 0.005]	−0.008	[−0.009, 0.007]	0.005	[−0.005, 0.006]	0.123 *	[0.003, 0.022]
Presence of bus stops	−0.038	[−0.015, 0.002]	−0.048 *	[−0.016, −0.000]	0.017	[−0.008, 0.010]	−0.039	[−0.015, 0.004]	−0.062	[−0.014, 0.002]	−0.083	[−0.021, 0.004]
Company density	−0.006	[−0.000, 0.000]	−0.011	[−0.000, 0.000]	0.035	[−0.000, 0.000]	−0.029	[−0.000, 0.000]	0.011	[−0.000, 0.000]	0.005	[−0.000, 0.000]
Presence of schools	−0.029	[−0.014, 0.005]	−0.033	[−0.014, 0.004]	−0.011	[−0.010, 0.008]	−0.026	[−0.013, 0.006]	−0.009	[−0.008, 0.007]	0.027	[−0.010, 0.016]
Socio−economic attributes												
Age	−0.222	[−0.011, 0.009]	−0.158	[−0.011, 0.009]	4.162	[−0.001, 0.021]	−0.625	[−0.015, 0.009]	−1.534	[−0.016, 0.005]	1.674	[−0.013, 0.027]
Education	−0.040	[−0.002, 0.001]	−0.042	[−0.002, 0.000]	0.172	[−0.000, 0.003]	−0.061	[−0.003, 0.001]	0.047	[−0.001, 0.002]	0.109	[−0.002, 0.005]
Employment	−0.019	[−0.008, 0.003]	−0.020	[−0.009, 0.003]	0.143 *	[0.001, 0.015]	−0.043	[−0.014, 0.003]	0.080 *	[0.001, 0.013]	−0.089	[−0.020, 0.003]
Married	0.060 *	[0.001, 0.026]	0.061 *	[0.002, 0.026]	0.100	[−0.014, 0.029]	−0.045	[−0.030, 0.013]	0.120 *	[0.000, 0.029]	0.088	[−0.006, 0.036]
Household size	0.015	[−0.002, 0.004]	0.015	[−0.002, 0.004]	0.135	[−0.002, 0.008]	0.067	[−0.001, 0.007]	0.080	[−0.000, 0.005]	−0.139 *	[−0.009, −0.001]
Household income	−0.023	[−0.003, 0.000]	−0.015	[−0.002, 0.001]	−0.035	[−0.003, 0.002]	−0.010	[−0.003, 0.002]	−0.036	[−0.003, 0.001]	−0.044	[−0.003, 0.001]
Wave												
2004 (ref.)												
2006	0.064	[−0.011, 0.032]	0.051	[−0.012, 0.030]	−0.276	[−0.039, 0.001]	0.047	[−0.020, 0.032]	0.164	[−0.007, 0.039]	−0.040	[−0.047, 0.039]
2009	0.087	[−0.037, 0.066]	0.062	[−0.041, 0.062]	−0.688	[−0.101, 0.012]	0.111	[−0.046, 0.076]	0.322	[−0.023, 0.085]	−0.285	[−0.130, 0.070]
2011	0.140	[−0.047, 0.094]	0.120	[−0.049, 0.090]	−0.888	[−0.136, 0.012]	0.211	[−0.057, 0.112]	0.428	[−0.034, 0.117]	−0.345	[−0.175, 0.104]
Attitudes												
Healthy diet	0.004	[−0.005, 0.006]	0.004	[−0.005, 0.006]	0.042	[−0.007, 0.012]	0.081 **	[0.003, 0.018]	−0.048	[−0.013, 0.005]	0.007	[−0.015, 0.016]
Physically active	−0.003	[−0.006, 0.005]	−0.002	[−0.006, 0.005]	−0.082	[−0.014, 0.004]	−0.037	[−0.014, 0.004]	0.007	[−0.005, 0.007]	−0.020	[−0.016, 0.012]
*N/N*_neighborhood	5479/69		5479/69		651/69		2196/69		1525/68		1107/69	
LL	9210.7		9236.0		1869.3		3998.6		3454.5		2403.6	
AIC	−18,383.4		−18,424.0		−3690.5		−7949.3		−6863.0		−4761.1	
BIC	−18,257.9		−18,265.3		−3583.0		−7812.6		−6740.4		−4645.9	

95% confidence intervals in brackets, * *p* < 0.05, ** *p* < 0.01, *** *p* < 0.001. ^a^ We also tested the cut-off at 0.85 for women and the results of significance and sign are the same with cut-off at 0.8.

**Table 3:** Standardized estimates of the effects of built environment elements and mediators on WHR, and the estimates of built environment elements on mediators.

	Continuous Population Density		Categorical Population Density		Mediators			
					WHR		Physical Activity Duration	
	Beta	CI	Beta	CI	Beta	CI	Beta	CI
Population density (Cont.)	0.034	[−0.001,0.003]			0.029	[−0.001,0.003]	−0.078 **	[−0.132, −0.025]
Population density								
0~0.1			0.087 *	[0.003, 0.023]				
0.1~0.5			0.027	[−0.003, 0.012]				
0.5~1 (ref.)								
1~1.5			−0.059	[−0.038, 0.003]				
1.5~2			−0.040 ***	[−0.033, −0.009]				
2~2.5			−0.029 *	[−0.024, −0.003]				
>2.5			0.052 **	[0.004, 0.028]				
Food environment								
Grocery density	−0.046 **	[−0.000, −0.000]	−0.050 ***	[−0.000, −0.000]	−0.046 **	[−0.000, −0.000]	0.028 **	[0.001, 0.003]
Restaurant types	0.072 *	[0.001, 0.008]	0.075 **	[0.001, 0.008]	0.068 *	[0.001, 0.008]	−0.015	[−0.166, 0.086]
Physical activity environment								
Presence of parks	0.014	[−0.006, 0.011]	0.015	[−0.006, 0.011]	0.018	[−0.006, 0.012]	−0.020	[−0.409, 0.134]
Presence of bus stops	−0.042	[−0.016, 0.002]	−0.052 *	[−0.017, −0.001]	−0.037	[−0.015, 0.002]	0.040	[−0.079, 0.636]
Company density	−0.009	[−0.000, 0.000]	−0.014	[−0.000, 0.000]	−0.007	[−0.000, 0.000]	−0.018	[−0.003, 0.001]
Presence of schools	−0.035	[−0.015, 0.004]	−0.038	[−0.015, 0.003]	−0.027	[−0.014, 0.006]	0.033	[−0.126, 0.559]
Socio−economic attributes								
Age	−0.261	[−0.011, 0.008]	−0.180	[−0.011, 0.009]	−0.235	[−0.011, 0.008]	−0.359	[−0.630, 0.447]
Education	−0.039	[−0.002, 0.001]	−0.043	[−0.002, 0.000]	−0.039	[−0.002, 0.001]	0.016	[−0.046, 0.071]
Employment	−0.020	[−0.009, 0.003]	−0.020	[−0.009, 0.003]	−0.021	[−0.009, 0.003]	−0.050 *	[−0.571, −0.044]
Married	0.063 *	[0.001, 0.027]	0.065 *	[0.002, 0.027]	0.060 *	[0.001, 0.026]	0.006	[−0.459, 0.566]
Household size	0.015	[−0.002, 0.004]	0.014	[−0.002, 0.004]	0.013	[−0.002, 0.004]	−0.056 *	[−0.226, −0.029]
Household income	−0.021	[−0.003, 0.000]	−0.014	[−0.002, 0.001]	−0.022	[−0.003, 0.000]	0.021	[−0.016, 0.112]
Attitudes								
healthy diet	0.000	[−0.006, 0.006]	0.000	[−0.006, 0.006]	0.005	[−0.005, 0.007]	0.040	[−0.056, 0.579]
physically active	0.002	[−0.005, 0.006]	0.003	[−0.005, 0.006]	−0.003	[−0.006, 0.005]	0.017	[−0.238, 0.464]
Mediators								
Smoker	0.018	[−0.006, 0.012]	0.014	[−0.007, 0.011]				
Drinker	−0.023	[−0.010, 0.003]	−0.020	[−0.009, 0.003]				
Car ownership	0.038 *	[0.001, 0.019]	0.040 *	[0.001, 0.019]				
Sleep duration	−0.002	[−0.002, 0.002]	−0.005	[−0.002, 0.002]				
Physical activity duration	−0.034 **	[−0.001, −0.000]	−0.032 *	[−0.001, −0.000]	−0.035 **	[−0.001, −0.000]		
Sedentary duration	0.033	[−0.000, 0.000]	0.035 *	[0.000, 0.000]				
Sick	−0.007	[−0.008, 0.005]	−0.009	[−0.008, 0.004]				
Wave								
2004 (ref.)								
2006	0.063	[−0.010, 0.031]	0.050	[−0.012, 0.029]	0.064	[−0.010, 0.032]	0.017	[−0.909, 1.146]
2009	0.089	[−0.036, 0.066]	0.061	[−0.040, 0.061]	0.089	[−0.036, 0.066]	0.062	[−2.309, 3.182]
2011	0.141	[−0.045, 0.092]	0.117	[−0.048, 0.088]	0.145	[−0.045, 0.093]	0.137	[−2.757, 4.683]
*N/N*_neighborhood	5393/69		5393/69		5479/69		5479/69	
LL	9109		9136		9215		−12,086	
AIC	−18,165		−18,210		−18,390		24,210	
BIC	−17,994		−18,006		−18,258		24,336	

95% confidence intervals in brackets, * *p* < 0.05, ** *p* < 0.01, *** *p* < 0.001
